# Metformin-loaded J-AuPPS for infected diabetic wound treatment

**DOI:** 10.3389/fbioe.2026.1753425

**Published:** 2026-02-19

**Authors:** Fuli Yin, Lingkai Yang, Haojie Shan, Yuange Li, Yongfeng Zhou, Xiaowei Yu

**Affiliations:** 1 Shanghai Sixth People’s Hospital Affiliated to Shanghai Jiao Tong University School of Medicine, Shanghai, China; 2 School of Chemistry and Chemical Engineering Frontiers Science Center for Transformative Molecules, Shanghai Jiao Tong University, Shanghai, China

**Keywords:** angiogenesis, antibacterial, diabetic wound infection, Janus particles, photothermal therapy, synergistic therapy

## Abstract

**Introduction:**

Infected diabetic wounds pose a significant clinical challenge owing to the complex wound microenvironment. Various multifunctional synergistic therapeutic nanomaterials have been successfully developed to treat diabetic infected wounds.

**Objective:**

A Janus Au–porphyrin polymersome heterostructure loaded with metformin (Met@J-AuPPS) is constructed and presented.

**Methods:**

Porphyrin polymersome vesicles loaded with metformin (Met@PPS) was synthesized by supramolecular polymerization enhanced self-assembly. Metformin-loaded Janus gold-porphyrin polymersome (Met@J-AuPPS) was synthesized by photocatalytic synthesis method. Transmission electron microscope (TEM), scanning electron microscope (SEM), UV-vis absorption spectrum and ELISA experiment were used to detect the morphology, structure, photothermal properties and drug release properties of Met@J-AuPPS. The effects of Met@J-AuPPS on the proliferation and activity of HUVECs were detected by CCK-8 test, scratch test, Transwell test and tube formation test. The antibacterial and antibiofilm abilities of Met@J-AuPPS were detected by blood plate test, crystal violet staining, SEM of biofilm and bacterial live/dead staining. Imaging and histological staining were used to detect the efficacy of Met@J-AuPPS in treating diabetic wound infections *in vivo*.

**Results:**

Excellent photothermal antibacterial performance against gram-positive methicillin-resistant *Staphylococcus aureus* (MRSA), gram-negative extended-spectrum β-lactamases *Escherichia coli* (ESBL *E. coli*), and MRSA/ESBL *E. coli* biofilm is demonstrated by a strong non-centrosymmetric near-field enhancement (NFE) effect between Met@PPS and Au nanoparticles. Furthermore, the release of metformin under near-infrared (NIR) light promotes angiogenesis and tissue repair. The results therefore show that Met@J-AuPPS exhibits excellent antibacterial/biofilm and pro-angiogenic performance, with significantly enhanced therapeutic effects in infected wounds of diabetic rat models.

**Conclusion:**

This study presents an innovative therapeutic strategy for diabetic wound infections and demonstrates that Met@J-AuPPS has potential as a multifunctional and upgradeable nanoplatform for further biomedical applications.

## Introduction

Diabetes, the most prevalent chronic metabolic disease worldwide, places a significant burden on the healthcare systems and society (Tomic et al., 2022; Yazdanpanah et al., 2015). Diabetic foot ulcers (DFU), which affect 18.6 million people globally each year, are linked to decreased quality of life, increased healthcare utilization, and impaired physical activity (Armstrong et al., 2023; Hicks et al., 2016). Diabetic wounds create a fertile environment for bacterial colonization. The two main clinical strategies for treating DFU are surgical debridement and antibiotic therapy (Chen et al., 2023a; Senneville et al., 2023). However, the administration of antibiotics at high doses frequently results in severe side effects, including life-threatening complications from drug-resistant bacterial infections (Hussain et al., 2018; Aumiller and Dollahite, 2015). A study by Alvaro-Afonso et al. compared the clinical outcomes of DFU caused by gram-positive methicillin-resistant *Staphylococcus aureus* (MRSA) and gram-positive methicillin-sensitive *Staphylococcus aureus* (MSSA). Their results showed that patients with MRSA had longer mean healing time compared to infections caused by MSSA (Álvaro-Afonso et al., 2025). And patients with MRSA used more antibiotics during the 1-year follow-up in another study (Suludere et al., 2024). Furthermore, the process of creating new antibiotics is getting harder and harder (Farha et al., 2018). In addition, diabetic wounds heal more slowly than normal wounds owing to chronic inflammation, decreased angiogenesis, and granulation tissue production, which can potentially result in amputation. (Wang et al., 2021; Koh and DiPietro, 2011).

Therefore, a variety of multifunctional synergistic therapeutic nanomaterials, including silver nanoparticles (Choudhury et al., 2020; Pérez-Rafael et al., 2021), gold nanoparticles (Vijayakumar et al., 2019; Boomi et al., 2020), metal–organic frameworks (Liu et al., 2021; Zhang et al., 2022) and peptides (Krizsan et al., 2014), have been successfully produced to treat diabetic infected wounds in addition to standard therapy. Multifunctional nanoplatforms are appealing approaches for the treatment of infected diabetic wounds due to their strong antibacterial qualities and great efficacy in encouraging wound healing, as evidenced by the materials’ exceptional performance. (P et al., 2025).

Janus nanoparticles (JNPs) have demonstrated great potential in the biomedical field among these many nanomaterials because they combine the mechanical, photoelectronic, and magnetic properties of inorganic materials with the benefits of polymers, including biocompatibility, environmental responsiveness, and ease of processing (Qiu et al., 2021; Zhang et al., 2016; Liu et al., 2016; Liu et al., 2014; Cao et al., 2021). In our earlier study, we used a one-step photocatalytic synthesis approach to successfully create a Janus Au-porphyrin polymersome (J-AuPPS) heterostructure. This technique produced a high near-field enhancement (NFE) effect, which improves the intensity of the interfacial electric/thermal field and the absorption of near-infrared (NIR) light. Both *in vitro* and *in vivo*, J-AuPPS exhibits encouraging photothermal antibacterial and anti-biofilm action. (Chen et al., 2023b). The polymersome in the J-AuPPS can be loaded with medications, targeting compounds, and diagnostic chemicals, offering an effective and expandable nanoplatform for additional medical applications.

Metformin is the first-line anti-diabetic drug (LaMoia and Shulman, 2021). Early studies suggest that the liver is the major organ involved in the reduction of blood glucose levels by metformin (Foretz et al., 2023). However, increasing evidence points to other important roles (Foretz et al., 2023; Dutta et al., 2023), including cardiovascular protection (Luo et al., 2019), anti-cancer effects (Zakikhani et al., 2006), neuroprotective effects (Palleria et al., 2016), anti-aging effects (Chen et al., 2022), and anti-inflammatory effects (Liu et al., 2023). Through mitochondrial autophagy, metformin also lessens the damage that hyperglycemia causes to endothelial cells and prevents microvascular damage in high-glucose conditions (Niu et al., 2019). Using metformin-release hydrogel dressings, Liang et al. helped patients with type II diabetes heal foot wounds by lowering inflammation and increasing angiogenesis through wound closure, re-epithelialization, blood vessel and follicle regeneration, and collagen metabolism (Liang et al., 2022). *In vitro* and *in vivo*, Ruan et al. discovered that metformin stimulated angiogenesis by blocking YAP1/TAZ in endothelial cells, which promoted the expression of HIF-1α. (Ruan et al., 2023). The drug-bioactive material combination synergistically promotes wound healing; however, it remains unclear whether the combinatorial effect of metformin and J-AuPPS in infected chronic diabetic wound healing is additive or synergistic.

**SCHEME 1:**
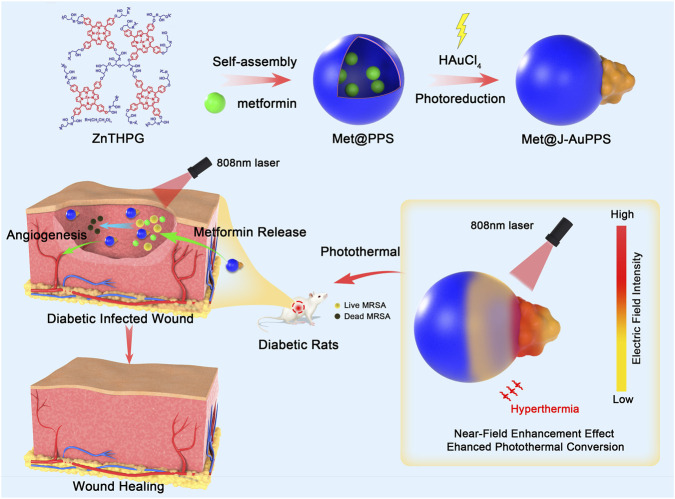
Schematic illustration of the preparation, electromagnetic NFE mechanism therapeutic effect of Met@J‐AuPPS.

Here, we report the construction of a Janus Au–porphyrin polymersome heterostructure loaded with metformin (Met@J-AuPPS) (Scheme 1). First, aqueous self-assembly of porphyrin hyperbranched polymers of 5,10,15,20-tetrakis (4-hydroxyphenyl) porphyrin Zinc (ZnTHPG) produced a porphyrin polymersome loaded with metformin (Met@PPS). Second, *in situ* photoreduction of Au ions followed by an Ostwald ripening process produced a unique Janus particle of Met@J-AuPPS, which is composed of a Met@PPS and an Au NP connected by the interconnecting interface. At the interface of Met@PPS and Au nanoparticles, a potent near-field enhancement (NFE) effect was produced, leading to both a larger electric/thermal field and better near-infrared absorption (Zhang et al., 2019). Met@J-AuPPS appears to display excellent photothermal antibacterial performance against gram-positive methicillin-resistant *Staphylococcus aureus* (MRSA), gram-negative extended-spectrum β-lactamases *Escherichia coli* (ESBL *E. coli*), and MRSA/ESBL *E. coli* biofilm *in vitro*. Met@J-AuPPS demonstrated clear angiogenic ability *in vitro* and markedly enhanced the migration and tube formation of human umbilical vein endothelial cells (HUVECs) upon near-infrared (NIR)-triggered drug release. Most importantly, Met@J-AuPPS Met@J-AuPPS demonstrated excellent promise in phototherapy for infected diabetic wounds. These results demonstrate that Met@J-AuPPS is bio-safe *in vivo* and is effective in eliminating MRSA biofilm on the wound under NIR irradiation. Synergistically, metformin can reduce inflammation, promote collagen secretion, and induce wound neovascularization.

## Methods

### Preparation of Met@J-AuPPS

The Met@PPS was synthesized following previous methods (Liu et al., 2020). Hydrophilic-hydrophobic interactions and supramolecular polymerization between porphyrin molecules were started by gradually adding water to the polymer’s solvent solution. Typically, 10 mg of polymer and 0.1/0.2/0.5 mg metformin were dissolved in 5 mL of DMF, and, after sonication for 10 min, filtered through a nylon filter with a pore size of 22 µg. Then, 10 mL of deionized water was slowly added, dropwise, to the above solution. This was followed by transfer to a dialysis bag with a molecular weight cut-off of 3,500 Da and dialysis in water for 48 h to remove DMF to obtain an aqueous solution of polymer vesicles. The volume of the assembly solution was used to calculate the vesicle concentration. Met@PPS aqueous solution (40 μg mL^−1^) was dispersed by ultrasonication for 10 min; then, HAuCl_4_ (10 mM, equivalent to 15 wt% Au) was added to the solution and stirred for 5 min in the dark, followed by reaction at 25 °C under visible light (λ > 420 nm, 300 W mercury lamp) for 1 h. The color of the solution deepened as the gold ions were reduced. After photoreaction, excess ions were removed by centrifugation and washing with deionized water for three times to obtain Janus-shaped Met@J-AuPPS.

### Scanning Electron Microscopy (SEM)

The sample preparation process was carried out as follows: the assembly solution (0.1 mg mL^-1^) was dropped on a clean silicon wafer, and, after air-drying, gold ions were sprayed on the surface of the sample. Then, SEM images were acquired with a Nova NanoSEM 450 field emission scanning electron microscope (FEI Corporation, United States).

### Transmission Electron Microscopy (TEM)

The sample preparation process was as follows: the assembly solution (0.1 mg mL^-1^) was dropped on a 200-mesh carbon film, and then air-dried for TEM photography on a Tecnai G2 biotype transmission electron microscope (Thermofisher, United States).

### UV-Vis absorption spectrum

The UV-Vis absorption spectra of polymers, assemblies, and composite structures were tested on a Lambda 35 UV-Vis absorption spectrometer (PerkinElmer, United States). The sample solution was dispersed in a 1 × 1 cm quartz cell and the test temperature was 298 K.

### ^1^H Nuclear Magnetic Resonance (^1^H NMR)

Five to 10 mg of sample was dissolved in 0.6 mL of deuterated reagent (DMSO-*d*_*6*_), and then tested with a AVANCE III HD 400 nuclear magnetic resonance spectrometer (Bruker, United States). DMSO or TMS was used as the internal standard, and the test temperature was 298 K.

### Gel Permeation Chromatography (GPC)

Gel permeation chromatography of ZnTHPG was performed on a HLC-8320GPC gel permeation chromatograph (Tosoh Corporation, Japan). A solution with a polymer concentration of 10 mg mL^-1^ was prepared. After dissolving and standing overnight, it was filtrated through a PTFE filter with a pore size of 0.22 µg. The test temperature was 298 K, DMF was the eluent, and the reference molecule was polystyrene.

### Dynamic Light Scattering (DLS)

The particle sizes of the self-assembled polymer vesicles and Met@AuPPS were characterized by DLS on a Nanoparticle Size and Zeta Potential Analyzer ZS90 (Malvern Instruments Ltd, UK). The measuring angle was 90° and test temperature was 298 K.

### Infrared thermal imaging

The thermal properties of PPS, J-AuPPS, and Met@J-AuPPS, as well as the thermal imaging effects of the samples *in vivo*, were captured by an infrared thermal imager (FOTRIC220 s, China).

### ELISA

The metformin (Met) ELISA Assay Kit (Zhenke, Shanghai) was used to determine the metformin content released after NIR irradiation of the Met@J-AuPPS solution. The sample wells were supplemented with 50 µL of Met@J-AuPPS solution irradiated by laser for different durations (1, 2, 3, 4, and 5 min), followed by the addition of working solution. The plate was covered with film and incubated at 37 °C for 15 min prior to measurement of OD values at a wavelength of 450 nm using the ELX‐800 absorbance microplate reader. The concentration of metformin in the sample was calculated based on the standard curve obtained from the standard products.

### Cytotoxicity evaluation

HUVECs (The cell lines present in this study were obtained from National Collection of Authenticated Cell Cultures) were used to determine the cytotoxicity of Met@J-AuPPS using the CCK-8 kit (Dojindo, Japan). Briefly, cells, at a concentration of 1 × 10^4^ cells/well, were cultured with Met@J-AuPPS (400 μg mL^−1^) in a 96-well-plate for 24, 48, and 72 h. PBS was set as a control group. After washing with fresh PBS twice, the cells were stained with 10% CCK-8 solution for 4 h. The relative cell density was determined by a microplate reader at a wavelength of 450 nm.

To investigate cellular damage by Met@J-AuPPS under NIR irradiation, 1 × 10^4^ cells/well HUVECs were co-incubated with 400 μg mL^-1^ Met@J-AuPPS, and then NIR-irradiated (1,000 mW cm^-2^) for 5 min. Next, the cells were rinsed with PBS twice and re-cultured with fresh culture medium. On day 1, day 2, and day 3, the cell viability was determined by CCK-8 assay, as described above.

### Cell scratch assay

For this assay, 1 × 10^6^ HUVECs per well were seeded in 6‐well plates. When the confluence of cells reached 80%, the 200 µL pipette tip was used to scrape the cells in a line to create wounds. Met, J-AuPPS, and Met@J-AuPPS were added to each well and irradiated with NIR (1,000 mW cm^-2^) for 5 min. Images were captured by inverted microscopy at 0 and 48 h after wounding. The migration area was quantified using ImageJ software.

### Transwell assay

For the transwell assay, 2 × 10^4^ HUVECs per well were seeded in the upper chamber of 24‐mm Transwell chambers (Corning #3412, United States) with 200 µL serum‐free DMEM medium. Met, J-AuPPS, and Met@J-AuPPS were added to each well and irradiated with NIR (1,000 mW cm^-2^) for 5 min. The lower chamber was filled with DMEM medium containing 10% FBS. After incubation for 24 h, cells in the upper chamber were fixed with 4% paraformaldehyde solution and stained with 0.1% crystal violet for 20 min. Subsequently, the chambers were air‐dried and images were captured. Migrated cells were quantified using ImageJ software.

### Tube formation assay

The effect of Met@J-AuPPS on the tube-forming ability of HUVECs was investigated. The matrigel was thawed overnight at 4 °C, and sterile tips and angiogenesis slides (Ibidi, Germany) were precooled. The next day, the matrigel was quickly added to the angiogenesis slides and placed in a cell incubator for 60 min at 37 °C. Then, 8 × 10^3^ HUVECs per well were seeded in angiogenesis slides. Next, Met, J-AuPPS, and Met@J-AuPPS were added to each well and irradiated with NIR (1,000 mW cm^-2^) for 5 min. Angiogenesis slides were observed at 4, 6, 8, 10, and 12 h, and photographed using an optical microscope. Tubes were quantified using ImageJ software.

### Spread plate method

Methicillin-resistant *S. aureus* (MRSA, ATCC43300) and extended-spectrum *β-*lactamase-producing *E. coli* (ESBL *E. coli*, ATCC35218) were selected as the two types of model bacteria. Single colonies were transplanted into tryptic soy broth (TSB) and grown at 37 °C overnight. The harvested cultures were centrifuged and adjusted to 10^8^ CFU mL^-1^. After that, 10 μL bacterial suspension was mixed with 1 mL PBS (as control), J-AuPPS (400 μg mL^-1^), or Met@J-AuPPS (400 μg mL^-1^) in centrifuge tubes with or without 808 nm NIR irradiation (1,000 mW cm^−2^) for 10 min. Then, 10 μL of serially diluted bacterial solution was spread on a sheep blood agar plate and incubated overnight at 37 °C. Next, the bacterial colonies were counted.

### Crystal violet staining

As-prepared bacterial solution was added into a 24-well plate and cultured for 48 h. The plate was washed with PBS, and J-AuPPS or Met@J-AuPPS were added to each well and irradiated with NIR (1,000 mW cm^-2^) for 10 min. After washing again, the biofilm was fixed with 4% paraformaldehyde solution for 10 min and stained with 0.1% crystal violet for 20 min. Then, 3% glacial acetic acid solution was used for decolorization of crystal violet in the biofilm. The OD value of each well was determined at 595 nm wavelength.

### Live/dead staining to assess bacterial viability

A sterile pure titanium disk was immersed into as-prepared bacterial solution and co-incubated for 48 h at 37 °C. Then, titanium was gently rinsed with aseptic PBS twice and re-immersed into PBS (control), J-AuPPS and Met@J-AuPPS under NIR laser irradiation (1,000 mW cm^-2^) for 10 min. Next, the titanium disk was rinsed with PBS twice. A Bacterial Live/Dead Staining Kit was used to visualize the ratio of live cells/dead cells in the biofilm. Briefly, as-prepared titanium disks were immersed in a 1 mL mixture of STYO9 and PI (1: 1,000 v/v in saline) in a 24-well-plate. After co-culturing for 30 min, the live/dead cells encapsulated in biofilm were observed with CLSM.

### SEM of biofilm

The as-treated titanium disks were fixed with 2.5% glutaraldehyde solution at 4 °C overnight, followed by sequential dehydration in a serial dilution of ethanol (30, 50, 70, 85, 90, 95% and 100%, v/v) for 10 min, according to standard procedures. Next, titanium disks were freeze-dried for 24 h prior to scanning electron microscopy.

### Treatment of diabetic infected wounds *in vivo*

All animal experimental procedures were performed in accordance with guidelines, and approved by the Ethics Committee of Shanghai Jiaotong University School of Medicine Affiliated Shanghai Sixth People’s Hospital (No.DWLL205-0010). A diabetic infected wound model was constructed using SD rats (∼250 g), and all rats were anesthetized by an intraperitoneal injection of pentobarbital sodium at a dose of 40 mg/kg. A diabetic rat model was established with Streptozocin (STZ) by intraperitoneal injection. A circular wound (1 cm) was created on the dorsal area of each rat after 2-week feeding. The wound was inoculated with MRSA bacteria (1 × 10^7^ CFU/mL), and a biofilm was formed after 2 days to construct a wound infection model in diabetic rats.

The rats were then randomly divided into eight groups: NIR (+) groups: PBS, Met, J-AuPPS, and Met@J-AuPPS groups; NIR (−) groups: PBS, Met, J-AuPPS, and Met@J-AuPPS groups. Next, 50 μL of samples were added on the wound with or without NIR laser irradiation (1,000 mW cm^−2^) for 5 min, and local temperature changes were recorded. The skin wounds of rats were observed and photographed on days 3, 6, 9, and 12. On day 6 and day 12, rats were euthanized for further analysis. Skin tissues as well as major organs, including heart, liver, spleen, lung, and kidney, were collected for H&E staining, Masson staining, Giemsa staining, and immunofluorescence staining.

### Statistical analysis

Prism nine software (GraphPad, US) and Origin 9 (OriginLab, US) were used for data analyses. The data were presented as the mean ± SD. T-test or one-way ANOVA was used for statistical analysis. A value of P < 0.05 was considered to indicate statistical significance, indicated as *P < 0.05, **P < 0.01, and ***P < 0.001.

## Results

### Preparation and Characterization of Met@J-AuPPS

First, ZnTHPG was synthesized according to a previous study (Liu et al., 2020). The ^1^H NMR spectrum of ZnTHPG is shown in Supplementary Figure S1. Subsequently, the assembly procedure was then started by gradually adding water to the ZnTHPG/N,N-dimethylformamide (DMF) solution of the polymer in order to begin supramolecular polymerization and hydrophilic-hydrophobic interactions between porphyrin molecules. During the synthesis of PPS, we added metformin to the polymer solution to prepare metformin-loaded PPS (Met@PPS). The proportions of metformin were 1%, 2%, and 5%, respectively.

**FIGURE 1:**
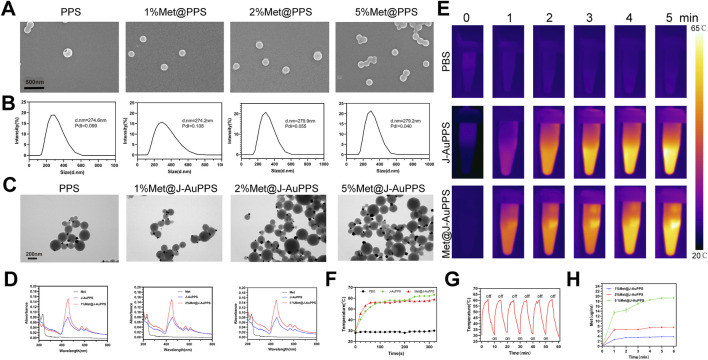
Preparation, characterization, photothermal and metformin release performances of Met@J‐AuPPS. **(A)** SEM images of PPS and Met@PPS; **(B)** DLS results of PPS and Met@PPS; **(C)** TEM images of J‐AuPPS and Met@J‐AuPPS; **(D)** UV–vis absorption spectra of J‐AuPPS and Met@J‐AuPPS; **(E)** Infrared thermal images and **(F)** temperature change of PBS, J‐AuPPS (400 μg mL^−1^) and Met@J‐AuPPS (400 μg mL^−1^) under an 808 nm laser (1000 mW cm^−2^); **(G)**The heating and cooling cycles of J‐AuPPS (400 μg mL^−1^, 1000 mW cm^−2^); **(H)** Metformin release curve of Met@J‐AuPPS (400 μg mL^−1^) under an 808 nm laser (1000 mW cm^−2^).

Transmission electron microscopy (TEM), scanning electron microscopy (SEM), and dynamic light scattering (DLS) were used to analyze the morphology and structure of the as-prepared PPS and Met@PPS(Figure 1A,B; Supplementary Figure S2). As shown in Figure 1, PPS, 1%Met@PPS, 2%Met@PPS, and 5%Met@PPS all formed smooth vesicle structures with uniform size. Some vesicles aggregated, which may have been due to the hydrophilic structure of the PPS vesicle surface. The DLS results showed that the vesicle size was similar between the four groups, indicating that the concentration of metformin had no significant effect on the uniformity and distribution of vesicle size. These results suggest that metformin does not interfere with the self-assembly of ZnTHPG. Therefore, we speculate that the properties of ZnTHPG loaded with metformin should be the same as those of hollow PPS, which provides the foundation for the synthesis and application of subsequent materials.

The J-AuPPS and Met@J-AuPPS heterostructures were then obtained by introducing HAuCl_4_ as an Au source into the PPS aqueous solution at 25 °C while being exposed to visible light. The TEM imaging validated the Janus nanostructure (Figure 1C). We counted more than 200 nanoparticles after the photoreaction. As can be seen from the statistical results, only one Au nanoparticle grew on most of the vesicles (Supplementary Figure S3). To verify whether the metformin was successfully loaded, we characterized materials by UV-Vis absorption spectroscopy (Figure 1D). The absorption peak of Met@J-AuPPS was significantly higher than that of J-AuPPS at a wavelength of 400–550 nm, which proved that the three groups of Met@J-AuPPS were well loaded with metformin, indicating the feasibility and reliability of it.

### Photothermal performance and metformin release of Met@J-AuPPS

Compared to other nanostructures, we have shown that J-AuPPS exhibits improved photothermal conversion efficiency (48.4%) and near-field enhancement effect (NFE) in the NIR spectrum (Chen et al., 2023b). According to the infrared thermal images, when exposed to an 808 nm laser, Met@J-AuPPS showed a comparable temperature increase from 29.5 °C to 59.8 °C to J-AuPPS (27.4 °C–63.1 °C) (Figure 1E,F). Additionally, after six cycles of heating and cooling, J-AuPPS and Met@J-AuPPS retained good photothermal characteristics (Figure 1G). These results confirm that, as expected, the loading of metformin does not adversely affect its original photothermal properties and photothermal conversion efficiency, and indicate that Met@J-AuPPS has satisfactory photothermal properties. Next, we tested the metformin release capacity of 1% Met@J-AuPPS, 2% Met@J-AuPPS, and 5% Met@J-AuPPS (Figure 1H). Met@J-AuPPS showed higher metformin release rate within 1 min, and almost all metformin was released within 5 min.

### Pro-angiogenesis ability *in vitro*

Good biocompatibility is a prerequisite for the application of Met@J-AuPPS in diabetic wound healing. The cytotoxicity of J-AuPPS to cells was comparatively low when the feeding concentration was less than 400 μg mL^−1^, in our previous research (Chen et al., 2023b). J-AuPPS and 1%/2%/5% Met@J-AuPPS (400 μg mL^−1^) were respectively co-cultured with HUVECs. CCK-8 assay revealed no significant difference in cell activity between the groups after 24, 48, and 72 h (Supplementary Figure S4A). Moreover, NIR-activated J-AuPPS and Initially, Met@J-AuPPS caused enormous cell death, although this effect was partly lost after being cultured for 2 and 3 days (Supplementary Figure S4B). These findings suggest that J-AuPPS and Met@J-AuPPS can cause damage to cells due to photothermal effects induced by NIR laser irradiation, but the damage is controlled and transient. More cells still maintain normal proliferative activity to eliminate this damage. These results demonstrate that Met@J-AuPPS has further utilization prospects in biological applications. Based on the above results, 5%Met@J-AuPPS(400 μg mL^−1^) was selected for follow-up studies.

**FIGURE 2:**
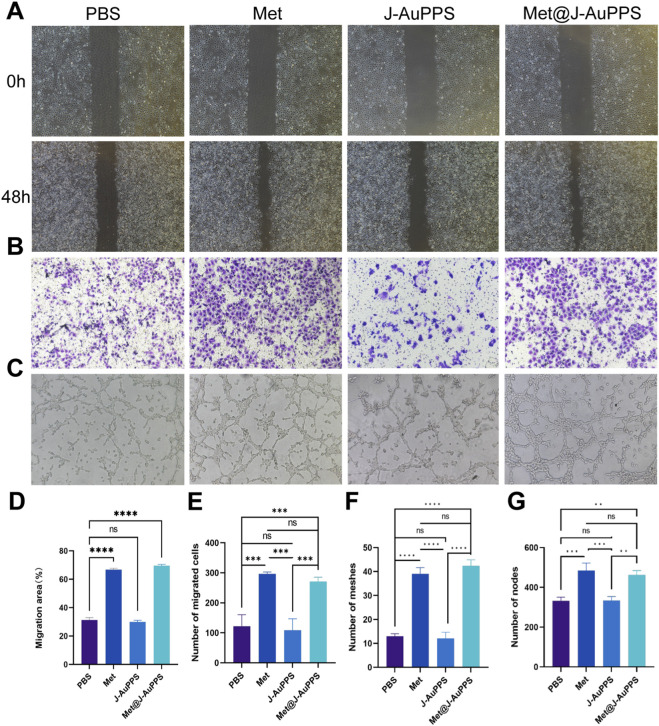
Pro-angiogenesis ability of Met@J-AuPPS in vitro. **(A)** Cell scratch test of HUVEC after co-incubated with J-AuPPS (400 μg mL^−1^) and Met@J-AuPPS (400 μg mL^−1^) for 48h; **(B)** Transwell migration test of HUVEC after co-incubated with J-AuPPS (400 μg mL^−1^) and Met@J-AuPPS (400 μg mL^−1^) for 24h; **(C)** Tube forming test of HUVEC after co-incubated with J-AuPPS (400 μg mL^−1^) and Met@J-AuPPS (400 μg mL^−1^) for 6h; **(D)** Quantitative analysis of migration area of HUVECs in cell scratch test (n=3, ****P < 0.0001); **(E)** Quantitative analysis of the number of migrating cells in Transwell migration test (n = 3, ***P < 0.001); Quantitative analysis of the number of meshes **(F)** and the number of nodes **(G)** formed in the tube forming test (n = 3, ***P < 0.001, ****P < 0.0001).

The effect of Met@J-AuPPS on HUVECs migration was evaluated by cell scratch test (Figure 2A). Compared with PBS and J-AuPPS, the number of migrated HUVECs after Met@J-AuPPS (NIR-triggered) treatment was significantly increased, showing similar effects as metformin treatment. The quantitative results of cell migration showed that the cell migration ratio of Met@J-AuPPS was significantly better than that of J-AuPPS (P < 0.01) (Figure 2D). The effect of Met@J-AuPPS was confirmed again by transwell migration test (Figures 2B,E). The ability of Met@J-AuPPS to induce tube formation by HUVECs was further tested, and the number of meshes and nodes was counted. As shown in Figures 2C F,G and only a small number of tubules were formed in the PBS group. Met@J-AuPPS induced the formation of more distinct and interconnected tubule structures by HUVECs. In summary, Met@J-AuPPS has ideal biocompatibility and good ability to promote angiogenesis *in vitro*.

### Antibacterial and anti-biofilm activity *in vitro*

**FIGURE 3:**
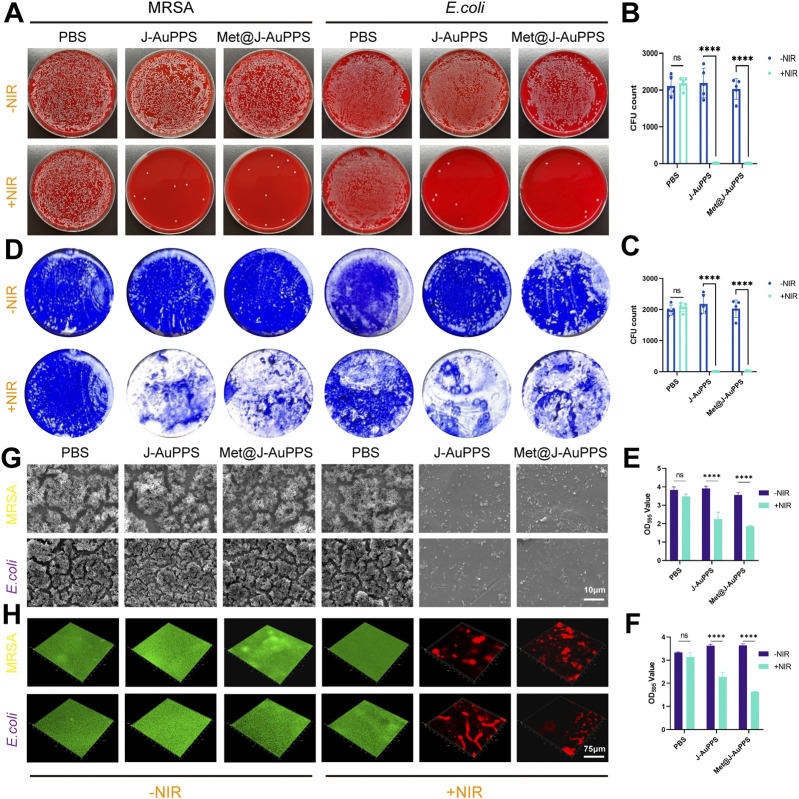
Antibacterial and anti-biofilm activity of Met@J-AuPPS in vitro. **(A)** Colony-forming units (CFUs) on sheep blood agar plates of MRSA and ESBL *E. coli* after treated with PBS, J-AuPPS (400 μg mL^−1^) and Met@J-AuPPS (400 μg mL^−1^) with or without NIR laser irradiation (808 nm, 1000 mW cm^−2^) for 10 min, respectively; **(B, C)** CFU count of MRSA **(B)** and ESBL *E. coli* **(C)** for the above groups. (n = 5, ****P < 0.0001); **(D)** Crystal violet staining of MRSA and *E.coli* biofilm after treated with PBS, J-AuPPS (400 μg mL^−1^) and Met@J-AuPPS (400 μg mL^−1^) with or without NIR laser irradiation (808 nm, 1000 mW cm^−2^) for 10 min, respectively; **(E,F)** OD595 value of MRSA **(E)** and *E. coli* **(F)** for the above groups (n = 5, **** p < 0.0001); **(G)** SEM images of MRSA/*E.coli*-biofilm on the surface of Ti disk in different groups; **(H)** CLSM images of MRSA and* E.coli* biofilm on Ti disk using Bacterial Live/Dead staining in each group (red: dead cells, green: live cells) after treated with PBS, J-AuPPS (400 μg mL^−1^) and Met@J-AuPPS (400 μg mL^−1^) with or without NIR laser irradiation (808 nm, 1000 mW cm^−2^) for 10 min, respectively.

Next, J-AuPPS and Met@J-AuPPS were evaluated for their antibacterial efficacy against MRSA and ESBL *E. coli*. The spread-plate method was used to assess the impact of J-AuPPS and Met@J-AuPPS on bacterial viability. As shown in Figures 3A–C, the NIR-activated J-AuPPS group nearly entirely scavenged MRSA or ESBL *E. coli* (bacterial viability <1%), indicating that PTT has a desirable antibacterial impact against microorganisms that are resistant to many drugs. Meanwhile, Met@J-AuPPS + NIR group showed similar results for antibacterial efficiency. The results reveal that the present nanomaterials kill both gram-negative and -positive bacteria by generating high local temperature through PTT, and metformin has no adverse effect on the PTT antibacterial properties.

Adhesion and colonization of bacteria further lead to the formation of biofilms. Antibiotic therapy and the host immune response are both highly tolerated by bacteria in biofilms (Yelin and Kishony, 2018). Therefore, the anti-biofilm properties of Met@J-AuPPS and J-AuPPS were assessed. Subsequently, following a 48-h biofilm-forming culture, MRSA and ESBL *E. coli* were exposed to NIR laser irradiation while being treated with J-AuPPS and Met@J-AuPPS. Using crystal violet staining, the biomass was examined to assess each sample’s anti-biofilm efficacy (Figures 3D–F). The biofilm elimination efficiency of the J-AuPPS and Met@J-AuPPS groups with NIR irradiation was 45.8% and 56.4%, respectively, according to the normalization of staining findings. Moreover, SEM was used to visualize the morphological changes of biofilms on the titanium (Ti) surface. The NIR-activated J-AuPPS and Met@J-AuPPS groups only had sporadic bacteria, but the control group had a dense bacterial cluster (Figure 3G). In addition, the bacterial live/dead (green/red) staining assay was used to assess the therapeutic efficacy of J-AuPPS and Met@J-AuPPS. The mature biofilms were thick and undamaged in control groups, which were stained green (Figure 3H). The NIR-activated J-AuPPS and Met@J-AuPPS groups, on the other hand, showed greater red fluorescence and a sparser biofilm, indicating that the biofilm structure was disrupted down and that the sealed bacteria were significantly reduced. These findings showed that the anti-biofilm properties of Met@J-AuPPS and NIR-activated J-AuPPS are comparable *in vitro*.

### NIR-triggered treatment in infected wound model of diabetic rats

**FIGURE 4:**
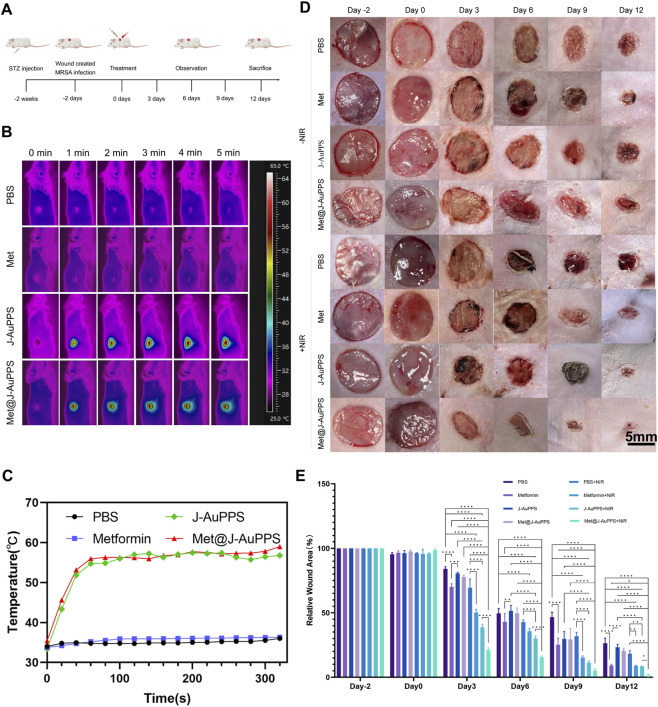
NIR-triggered treatment in infected wound model of diabetic rats. **(A)** Flow chart of Met@J-AuPPS treatment of infected wounds in diabetic rats. **(B)** Infrared thermal images and **(C)** temperature change of PBS, Met (20 μg mL^−1^), J-AuPPS (400 μg mL^−1^) and Met@J-AuPPS (400 μg mL^−1^) under an 808 nm laser (1000 mW cm^−2^) in the infected wound of diabetic rats. **(D)** Photos of biofilm infected wound of diabetic rats; **(E)** Quantitative analysis of relative wound area. (n = 5, *P < 0.05, **P < 0.01, ***P < 0.001,**** p < 0.0001).

Based on the results that Met@J-AuPPS demonstrated ideal *in vitro* biocompatibility and excellent *in vitro* antibacterial ability, the ability of Met@J-AuPPS to accelerate infected wound healing was evaluated by establishing a MRSA-infected full-thickness skin defect diabetic rat model. The progression is illustrated in Figure 4A. The treatments were divided into eight groups: PBS, Met, J-AuPPS, Met@J-AuPPS, PBS + NIR, Met + NIR, J-AuPPS + NIR, Met@J-AuPPS + NIR(Figures 4B,C). Wound photographs were taken on the 3rd, 6th, 9th, and 12th day to assess the degree of wound healing. The Met@J-AuPPS + NIR group showed the best healing status without visible scarring (Figure 4D). The relative wound area with time (Figure 4E) showed that the healing rates of wounds in the Met, Met + NIR, J-AuPPS + NIR, and Met@J-AuPPS + NIR groups were much faster than those in other groups. Notably, the relative wound area of the Met@J-AuPPS + NIR group was the smallest among all the groups at day 12.

**FIGURE 5:**
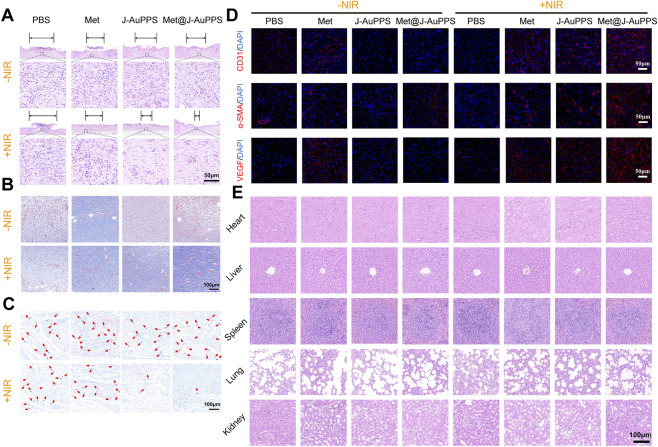
Figure 5. Wound tissue analysis in vitro. **(A)** HE and **(B)** Masson staining of MRSA biofilm infected wound of diabetic rats; **(C)** Giemsa staining of MRSA biofilm infected wound of diabetic rats (The red arrows: residue bacterial aggregates); **(D)** Immunofluorescence staining of CD31, α-SMA and VEGF of wound tissue; **(E)** HE staining of heart, liver, spleen, lung and kidney of diabetic rats.

These results were confirmed by H&E and Masson staining, which demonstrated the excellent performance of Met@J-AuPPS in accelerating diabetic wound healing (Figures 5A,B). More skin defects and inflammatory cells were found in the PBS, J-AuPPS, Met@J-AuPPS, and PBS + NIR groups. In comparison, more complete skin structure, fewer inflammatory cells, and more collagen fibers were found in the Met, Met + NIR, J-AuPPS + NIR, and Met@J-AuPPS + NIR groups, especially in the Met@J-AuPPS + NIR group. After the sixth day of wound healing, the bacterial residue was assessed by Giemsa staining (Figure 5C). Numerous bacterial aggregates were seen in the -NIR, PBS + NIR, and Met + NIR groups, suggesting a serious infection. The antibacterial properties of J-AuPPS and Met@J-AuPPS under NIR irradiation contributed to the nearly total elimination of the bacteria in the J-AuPPS + NIR and Met@J-AuPPS + NIR groups.

We then performed immunostaining with CD31/α-SMA/VEGF, which were angiogenesis markers. It was observed that the relative coverage area of CD31/α-SMA/VEGF was higher in the wound areas of the Met@J-AuPPS + NIR groups (Figure 5D), which was attributed to the angiogenic ability of metformin and the non-infectious environment. In contrast, other groups showed a lower relative coverage area, indicating an impaired angiogenic state with increased inflammation. Finally, the vital organs were harvested and stained with H&E for biocompatible assessment (Figure 5E).

## Discussion

In earlier work, we produced a Janus Au-porphyrin polymersome heterostructure which had higher photothermal conversion efficiency under 808 nm NIR laser irradiation compared to non-Janus core–particle Au–porphyrin polymersome nanostructure. To explore its further applications, in this study, a Janus Au–porphyrin polymersome heterostructure loaded with metformin was successfully constructed. We designed three proportions of metformin (1%/2%/5%) to determine the optimal drug loading concentration. Characterization of Met@J-AuPPS demonstrated that the loading of metformin had no adverse effect on the synthesis process and no significant change in the size and distribution of the polymers. Next, we detected photothermal performance and metformin release of Met@J-AuPPS. As expected, Met@J-AuPPS showed excellent photothermal conversion efficiency and similar rising temperature trend under 808 nm NIR laser irradiation compared to J-AuPPS. However, there was a slight difference between the final drug release concentration and the expected drug concentration, which was presumably caused by three reasons. First, during the preparation of the material, not all of the added metformin was loaded into PPS vesicles or some PPS vesicles were not assembled completely. Second, the detection was only carried out for 5 min by NIR light irradiation, and a few materials may not have been activated. Third, the detection sensitivity was limited, and there may have been experimental error.

In the process of diabetic wound repair, angiogenesis is crucial. Metformin was reported to stimulate angiogenesis (Liang et al., 2022; Ruan et al., 2023). Therfore, a coculture of Met@J-AuPPS and HUVEC cells was used to evaluate the effect of Met@J-AuPPS on the proliferation and migration of vascular cells. Results confirmed that Met@J-AuPPS can significantly promote the migration of HUVEC cells, and the migration of endothelial cells is also a necessary condition for the development of blood vessels. Further, we conducted tube formation experiments to explore the effect of Met@J-AuPPS on the angiogenesis ability of HUVEC. These results suggested that Met@J-AuPPS had good ability to promote angiogenesis *in vitro*.

In a hyperglycemic state, the wound is more susceptible to infection and become more difficult to heal. We found that the NIR-activated Met@J-AuPPS nearly entirely scavenged MRSA or ESBL *E. coli* (bacterial viability <1%) on the plate. However, adhesion and colonization of bacteria further lead to the formation of biofilms *in vivo*, causing more difficult to eliminate bacteria. Thus, it is necessary to test the effect of Met@J-AuPPS in removing biofilms. Crystal violet staining revealed that Met@J-AuPPS had excellent biofilm elimination efficiency. In addition, we used SEM and CLSM to conduct more detailed observations of biofilms. After treatment of Met@J-AuPPS, the destruction of bacterial structure, shrinkage and distortion of bacterial cells were observed. At the same time, large-scale bacterial death led to the removal of biofilms. These findings suggest that changes in the integrity of bacterial membranes were linked to the improved antibacterial action of nanostructures. As previously noted, PTT can result in membrane protein denaturation and excessive ROS accumulation within cells, both of which work together to worsen pathogenic bacteria’s eventual damage (Ran et al., 2021).

To explore he ability of Met@J-AuPPS to accelerate infected wound healing *in vivo*, we constructed MRSA-infected full-thickness skin defect diabetic rat model. The Met@J-AuPPS + NIR group showed the best healing status, due to the synergistic therapeutic effect of Met@J-AuPPS. The healing rates of wounds in the Met, Met + NIR, and J-AuPPS + NIR groups were also much faster than those in other groups, which indicated that photothermal antibacteria and drug-promoting angiogenesis had a positive impact on the repair of diabetic infected wounds, respectively. In addition, the wound healing of J-AuPPS + NIR group were faster than that in Met and Met + NIR groups. It suggested that the efficacy of metformin could not be realized unless the infection was effectively controlled. In the process of wound repair, collagen metabolism also plays an important role. More collagen fibers were found in the Met@J-AuPPS + NIR group and it often indicated better mechanical strength of the tissue after healing. Furthermore, the staining of CD31, α-SMA and VEGF was used to analyze the regeneration of blood vessels. CD31 can represent newborn blood vessels in the wound, while α-SMA represents mature blood vessels (Ma et al., 2020), and VEGF is critical to neovascularization in numerous tissues under physiological and pathological conditions (Vempati et al., 2014). In this study, the relative coverage area of CD31/α-SMA/VEGF was higher in the wound areas of the Met@J-AuPPS + NIR groups. We speculated that it was attributed to the angiogenic ability of metformin and also the non-infectious environment. Bacterial infection suppressed expression of VEGF, while metformin promoted it. More molecular mechanisms need to be further investigated.

There are still many limitations in this research. First, there is a lack of in-depth research on molecular mechanisms of the antibacterial, anti-biomembrane, regulation of HUVEC, and regulation of the immune system; Second, the period of study *in vivo* is short. We cannot predict the long-term occurrence of complications; Third, more biosafety studies such as RNA-seq test are needed to ensure clinical translation.

## Conclusion

In this study, a Janus Au–porphyrin polymersome heterostructure loaded with metformin was successfully constructed, with similar photothermal properties under 808 nm NIR laser irradiation compared with J-AuPPS and excellent photothermal antibacterial and anti-biofilm activities *in vitro*. Furthermore, the present heterostructure has ideal biocompatibility and good ability to promote angiogenesis *in vitro*, with significantly enhanced therapeutic effects observed in a diabetic rat wound model. These findings show that Met@J-AuPPS is an adaptable and upgradeable nanoplatform that can be loaded with additional medications, targeting molecules, or diagnostic tools for use in additional biomedical applications.

## Data Availability

The original contributions presented in the study are included in the article/Supplementary Material, further inquiries can be directed to the corresponding authors.
