# Supplementary material for: Metformin-loaded J-AuPPS for infected diabetic wound treatment
Source: Front Bioeng Biotechnol. 2026 Feb 19;14:1753425. doi: 10.3389/fbioe.2026.1753425 (PMC12960594; doi:10.3389/fbioe.2026.1753425)
Supplement: Supplementary file 1 [file DataSheet1.docx]

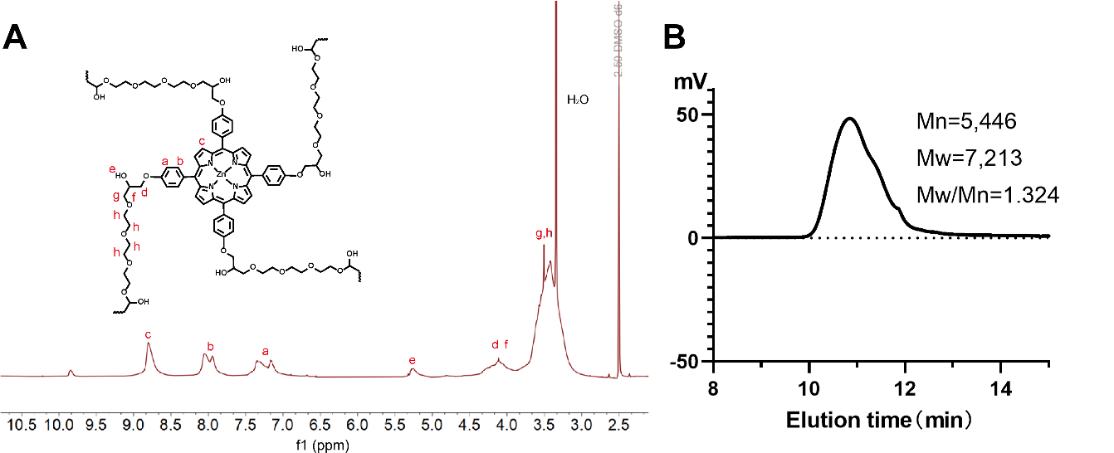


Figure S1. Characterizations of ZnTHPG. (A) 1H NMR spectrum of ZnTHPG (DMSO-*d6*, 25℃); (B)GPC curve of ZnTHPG (DMF as eluent and PS as standard sample, 25℃).


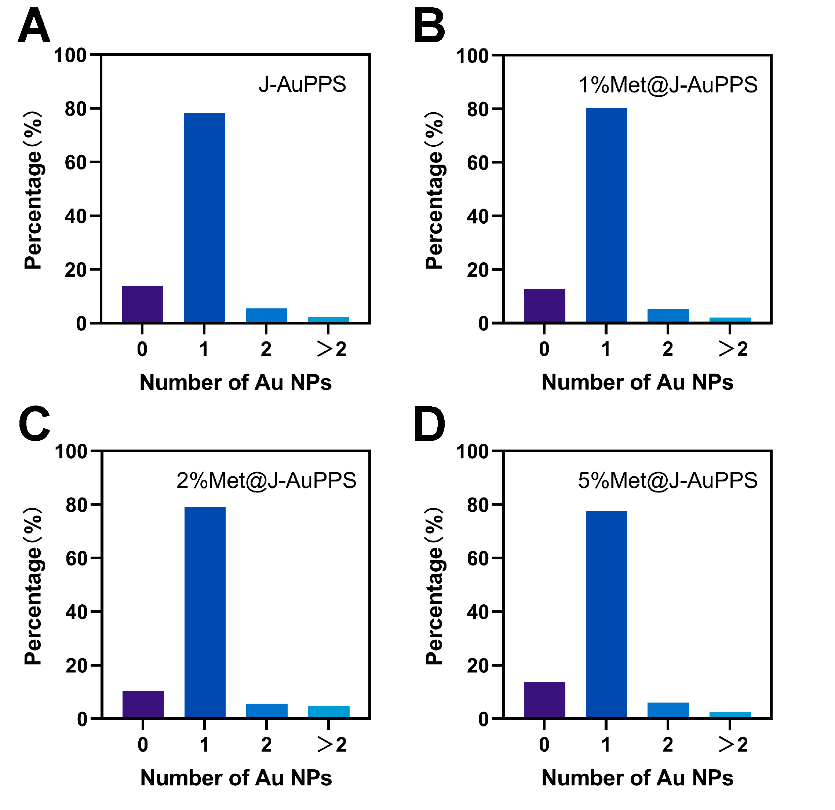


Figure S2. Statistical results of J-AuPPS(A), 1% Met@J-AuPPS(B), 2% Met@J-AuPPS(C), and5% Met@J-AuPPS(D) loaded with different numbers of Au NPs.


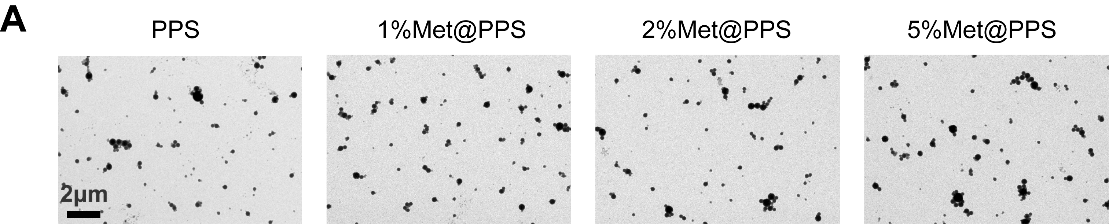


Figure S3. TEM of PPS, 1% Met@J-AuPPS, 2% Met@J-AuPPS, and 5% Met@J-AuPPS.


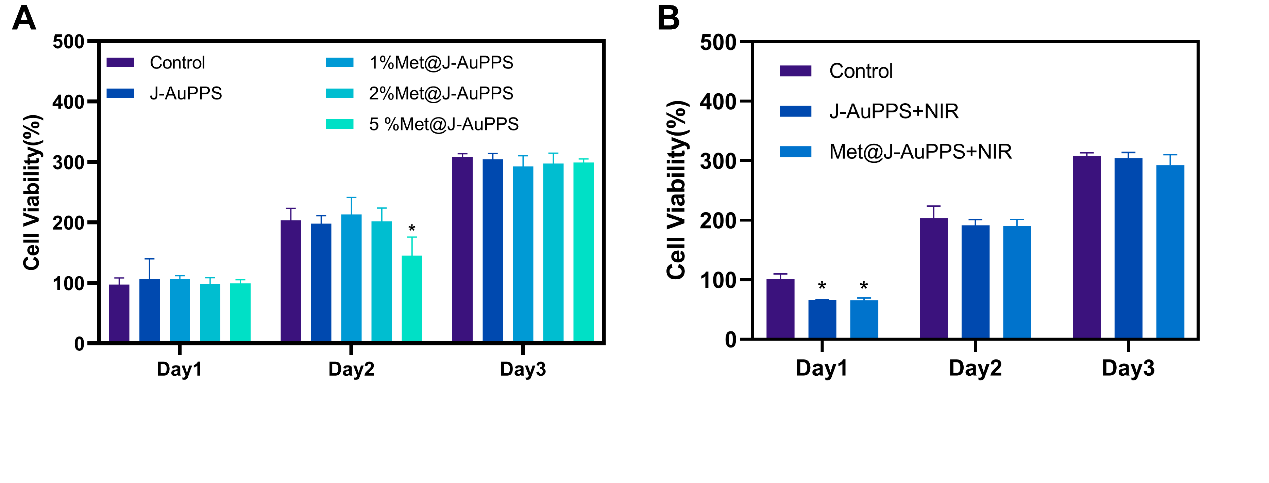


Figure S4. Cytotoxic evaluation of J-AuPPS. (A) Assessment of cell viability by CCK-8 assay after co-incubated with J-AuPPS (400 µg mL^−1^) and 1%/2%/5% Met@J-AuPPS (400 µg mL^−1^) on day 1 day 2 and day 3, (n = 3, *P﹤0.05); (B) Cell viability of HUVEC cells in day 1, day 2 and day 3 after treated by J-AuPPS (400 μg/mL) and 5% Met@J-AuPPS (400 µg mL^−1^) under NIR (808 nm, 1000 mW cm^2^) for 5 min, (n = 3, *P﹤0.05).
